# The Effects of Low-Dose Irradiation on Human Saliva: A Surface-Enhanced Raman Spectroscopy Study

**DOI:** 10.3390/diagnostics9030101

**Published:** 2019-08-22

**Authors:** Ioana Maria Colceriu-Șimon, Mihaela Hedeșiu, Valentin Toma, Gabriel Armencea, Alin Moldovan, Gabriela Știufiuc, Bogdan Culic, Viorica Țărmure, Cristian Dinu, Ioana Berindan-Neagoe, Rareș Ionuț Știufiuc, Mihaela Băciuț

**Affiliations:** 1 Department of Orthodontics and Dentofacial Orthopedics, “Iuliu Hațieganu” University of Medicine and Pharmacy, 400001 Cluj-Napoca, Romania; 2 Department of Oral Radiology, “Iuliu Hațieganu” University of Medicine and Pharmacy, 400006 Cluj-Napoca, Romania; 3 MedFuture Research Center for Advanced Medicine, 400337 Cluj-Napoca, Romania; 4 Department of Oral and Maxillofacial Surgery, “Iuliu Hațieganu” University of Medicine and Pharmacy, 400029 Cluj-Napoca, Romania; 5 Faculty of Physics, “Babeș-Bolyai” University, 400084 Cluj-Napoca, Romania; 6 Department of Prosthetic Dentistry and Dental Materials, “Iuliu Hațieganu” University of Medicine and Pharmacy, 400006 Cluj Napoca, Romania; 7 Research Center for Functional Genomics, Biomedicine and Translational Medicine, “Iuliu Hațieganu” University of Medicine and Pharmacy, 400337 Cluj-Napoca, Romania; 8 Department of Functional Genomics and Experimental Pathology, Oncological Institute “Prof. Dr. Ion Chiricuţă”, 400015 Cluj-Napoca, Romania; 9 Department of Pharmaceutical Physics-Biophysics, “Iuliu Hațieganu” University of Medicine and Pharmacy, 400349 Cluj-Napoca, Romania; 10 Department of Implantology and Maxillofacial Surgery, “Iuliu Hatieganu” University of Medicine and Pharmacy, 400033 Cluj-Napoca, Romania

**Keywords:** ionizing radiation, pediatric patients, Raman spectroscopy, thiocyanate

## Abstract

Biological effects of low-dose ionizing radiation (IR) have been unclear until now. Saliva, because of the ease of collection, could be valuable in studying low-dose IR effects by means of surface-enhanced Raman spectroscopy (SERS). The objective of our study was to compare the salivary SER spectra recorded before and after low-dose IR exposure in the case of pediatric patients (PP). Unstimulated saliva was collected from ten PP before and after irradiation with a cone beam computed tomography (CBCT) machine used for diagnostic purposes. The SERS measurements have been recorded on dried saliva samples using a solid nanosilver plasmonic substrate synthesized using an original method developed in our laboratory. The experimental results showed that salivary SER spectra are dominated by three vibrational bands (441,735 and 2107 cm^−1^) that can be assigned to bending and stretching vibrations of salivary thiocyanate (SCN-). After exposure, an immediate increase of vibrational bands assigned to SCN- has been recorded in the case of all samples, probably as a result of IR interaction with oral cavity. This finding suggests that SCN- could be used as a valuable biomarker for the detection and identification of low-dose radiation effects.

## 1. Introduction

Ionizing radiation (IR) is responsible for different biological effects that are strongly dependent on radiation type, exposure patterns and dose levels [1]. While the deterministic and stochastic effects of high-dose IR exposures have been widely reported in the literature [2,3], the risks of low-dose radiation still remain an important scientific open question that needs to be addressed. For instance, it has been shown that stochastic carcinogenic effects could occur as a consequence of low levels of radiation exposure with a cumulative risk over time [4].

Cone beam computed tomography (CBCT) is a low-dose radiological examination that is able to improve the diagnosis and treatment outcome in many fields of dentistry, including orthodontics and dentofacial orthopedics [5,6]. The study of the biological effects induced by CBCT exposures could represent the missing link in our understanding of low-dose radiation effects on human health, especially in the pediatric population (PP) which is more radiosensitive and has a longer lifespan. As such, the cumulative stochastic effects need to be properly evaluated [7].

In the literature there are very few molecular epidemiological studies capable of answering questions related to the effects of low doses of IR, such as individual radiosensitivity, noncancerous effects, and tissue sensitivity [8]. Moreover, a limited number of salivary biomarkers have been identified for IR research purposes. Among them none is able to accurately describe the effects of moderate doses of IR exposures [9]. So far, most of the low-dose IR biomarkers have been searched for in blood, cells, or tissue samples, but the development of simple and non-invasive methods for the study of biomolecular effects induced by low-dose radiation is of foremost importance [10].

In recent years, the use of saliva as a diagnostic tool has gained considerable attention. As a diagnostic fluid for monitoring oral and systemic health, saliva offers superiority over blood by being non-invasive to collect, easy to store, and cost-effective for large population screening [10]. The salivary glands are often within the primary CBCT radiation beam, receiving high radiation per organ dose [11]. Therefore, the salivary secretion could be used for specific biomarker identification in the case of low-dose radiation exposure, especially in children, for whom blood sampling is difficult [12].

Raman spectroscopy (RS) is a spectroscopic technique based on inelastic scattering of low-intensity, monochromatic laser light by the vibrating atoms of the sample under investigation [13]. The use of RS for biomolecular research became very popular since it is a non-destructive method capable to investigate different types of biosamples (tissues, live cells, biofluids), with little to no sample preparation [14]. Recently, it has been shown that significant changes in the Raman spectra have been detected in the case of irradiated dental pulp stem cells after repeated CBCT low-dose exposure [15]. New developments in Raman techniques, such as surface enhanced Raman spectroscopy (SERS), allowed major improvements in analyzing different types of biofluids [16]. SERS is a surface-sensitive technique that enhances the Raman scattering process for molecules situated in the immediate vicinity of plasmonic nanostructures [17]. It has been successfully employed for analyzing body fluids (blood plasma, serum, urine, saliva) and the results are very promising, especially in the case of cancer detection [16,18,19,20,21,22]. In a very recent study Stefancu et al. have shown that SERS analysis performed on saliva samples, combined with a principal component analysis-linear discriminant analysis (PCA-LDA), can be successfully employed for diagnosing Sjogren’s syndrome [23]. These results suggest that salivary biofluid could be used for studying low-dose radiation effects by means of SERS, even though in the scientific literature this type of research has not been reported up to now.

Thiocyanate (SCN-) is a biomolecule that acts as a natural antioxidant in the immune system and it was found in different types of human extracellular body fluids, including saliva [24]. So far, its presence has been detected by means of high-performance liquid chromatography (HPLC), spectrophotometry, ion chromatography-tandem mass spectrometry (IC-MS/MS), and ion chromatography [25,26,27,28,29].

Given its affinity for gold and silver plasmonic substrates, SERS can represent an alternative technique for SCN- detection in saliva, with high sensitivity and specificity. Moreover, SERS allows a precise SCN- identification without any interference from other coexisting anions since the C≡N stretching vibration presents a very strong vibrational band around 2100 cm^−1^, seldom overlaid by other vibrational modes [30]. It has also been reported that salivary thiocyanate levels can be used as biomarkers for smoker’s identification, since its value is much higher with respect to nonsmokers [31,32].

In this study we propose the use of salivary thiocyanate as a biomarker for evaluating the effects of low-dose radiation in PP. We have performed surface-enhanced Raman measurements on human saliva collected from children before and after CBCT exposure by using a solid plasmonic substrate developed in our laboratory. The evolution of the vibrational bands assigned to SCN- and of other vibrational bands upon CBCT exposure were monitored and estimated by comparing the SER spectra recorded before and after IR exposure. To the best of our knowledge this is the first time such an approach has been proposed in the scientific literature.

## 2. Materials and Methods

### 2.1. Saliva Sampling

The study was approved by the Clinical Research and Ethics Committee of the University of Medicine and Pharmacy, “Iuliu Hațieganu”, Romania (ethical approval number 282, ethical approval data 11 July 2017). Ten healthy patients aged 12–17 that underwent a CBCT examination for orthodontic treatment planning were recruited for this study. The patients included in this study were not taking any specific medication, were not smokers and had good oral hygiene. The written informed consent was signed by the parents of every patient.

Patients were exposed to radiation with a low-dose ProMax 3D CBCT machine (Planmeca, Finland) using the following protocol: large field of view (FOV), 83 kilovolts (kV) and 54 milliampere seconds (mA∙s). The effective doses of CBCT exposure for children were calculated based on the Monte Carlo simulation method with the correction factor for age and examination protocol that was applied, being between 174 and 256 µSv [33]. The variables of the patients irradiated with the large field of view CBCT, the irradiation duration and the effective irradiation dose are presented in Table S1.

Unstimulated, whole saliva was collected from each patient 30 min before and 30 min after CBCT exposure. Subjects did not eat or drink for 30 min before and after irradiation. Saliva samples were collected using the passive drool method [34]. Collection devices specifically designed to improve volume collection and to increase participant compliance (SalivaBio’s 2 mL cryovials and the Saliva Collection Aid (Salimetrics, State College, PA, USA)) were used in the present study. Immediately after collection, the saliva samples were frozen and stored at −25 °C for maximum three days. Before the measurements, the saliva samples were centrifuged at 805× *g* for 60 s in order to remove food debris and the supernatant was stored at −80 °C until further analysis. The supernatant was removed from the BioSaliva Passive Drool device and a small amount (~1 µL) was used for Raman/SERS measurements. No other filtering methods or treatments have been performed prior to Raman/SERS measurements.

### 2.2. Synthesis of Silver Nanoparticles (NPs)

Silver nitrate (AgNO_3_) used for synthesis of silver colloids was purchased from Roth GmbH, Karsruhe, Germany. The aqueous colloidal solution was prepared using ultrapure water (18.2 MΩ×cm, Chorus PureLab Elga, UK) according to the procedure proposed by Leopold and Lendl [18]. Briefly, 10.5 mg of hydroxylamine hydrochloride and 12 mg of sodium hydroxide were dissolved in 90 mL of ultrapurified water. In a second bottle 17 mg of AgNO_3_ were dissolved in 10 mL of ultrapurified water and the obtained solution was quickly poured in the previous one under vigorous stirring. The color of the final solution quickly changed from colorless to brown and finally to yellowish grey in the course of 5 min. At the end of the synthesis procedure, the colloid was rested for 24 h at room temperature before its purification and concentrated 10 times using the tangential flow filtration method (TFF) proposed by Dorney et al. [35].

### 2.3. SERS Substrate Preparation

Calcium fluoride (CaF_2_) polished glass coverslips of 25 mm diameter and 1 mm thickness (Crystran Limited, Poole, UK) have been used as port-probes for the synthesis of solid plasmonic SERS substrates. Prior to any measurement the coverslip was cleaned by using a standard plasma cleaning procedure [36]. The SERS substrate was produced by pouring very small amounts (~1 µL) of the silver colloid directly on the preheated glass surface (at 40–50 °C) and dried for 90 s. Finally, the CaF_2_ glass was left at room temperature for 30 min followed by the deposition of 1 µL of saliva samples (control and CBCT irradiated) directly on the solid SERS spots. The Raman measurements were performed on small amounts (~1 µL) of analytes, drop casted and dried directly on CaF_2_ glass (standard drop coating method).

### 2.4. UV–VIS Absorption Measurements

The VIS absorption spectrum of silver colloid was acquired using a T92+ UV–VIS spectrophotometer from PG Instruments, UK. The absorption curves have been recorded on standard quartz cells at room temperature, over a spectral range between 300 nm and 800 nm. The spectral resolution has been set at 2 nm.

### 2.5. Transmission Electron Microscope (TEM) Measurements

For a comprehensive characterization of silver NPs electron microscopy measurements were performed on a HT7700 (Hitachi, Japan) transmission electron microscope (TEM) operating at 100 kV, using the high-resolution operation mode (spot size ~0.60 µm). Colloidal samples were deposited on carbon films on top of copper grids for 5 min followed by the removing of excess and 15 min drying time.

### 2.6. Atomic Force Microscopy (AFM) Measurements

Atomic force microscopy (AFM) experiments have been performed under ambient conditions using a NT-MDT NTegra Vita system mounted on an inverted Olympus IX73 optical microscope (Olympus Corp., Tokyo, Japan. The measurements were performed in semi-contact mode using Si_3_N_3_ tips (NT-MDT) having a resonant frequency of 235 kHz and a nominal force constant of 12 N/m. The typical curvature radius of the tips is ~10 nm. The images were recorded on different regions of the solid plasmonic substrates in topographic and phase contrast mode. Prior to any measurements the quality of the AFM tips has been tested on standard topographic gratings provided by NT-MDT.

### 2.7. Raman/SERS Measurements and Data Collection

The Raman and SERS spectra were recorded using a Renishaw™ inVia Reflex Raman confocal multilaser spectrometer having a spectral resolution of 0.5 cm^−1^. The wavelength calibration was performed using an internal silicon reference. All the spectra presented in this paper were acquired using a 100× (N.A = 0.85) objective. A 785 nm diode laser (Renishaw, UK) was used for excitation. The laser power (measured at the sample surface) was ~65 mW for Raman measurements and ~1.95 mW for SERS ones. The acquisition time was set to 10 s. The spectrograph was equipped with a 1200 lines/mm grating and a charge coupled device camera (CCD). WiRE 4.2 software (Renishaw plc, Gloucestershire, UK) was used for data collection and spectral pre-processing, including cosmic ray removal and baseline correction. The latter one was applied to all spectra in order to eliminate the fluorescence background. Each spectrum was an average of 30 spectral acquisitions from different positions of the whole dried sample area.

## 3. Results and Discussion

### 3.1. Synthesis and Characterization of Solid SERS Substrates

Colloidal silver NPs have been widely used as plasmonic substrates for SERS measurements performed on liquid biological fluids (including saliva) [20,37]. The major drawback of this method is the unspecific adsorption of large biomolecules (such as proteins) present in large quantities in the biofluids, leading to the formation of a “protein corona”. This hinders NPs aggregation (due to a steric repulsion effect) and makes the adsorption of small biomolecules to the metallic substrate almost impossible [38,39]. As a result, the SER spectra recorded in these conditions are largely dominated by proteins, making the identification of small biomarkers by means of SERS difficult to achieve. In order to overcome this major obstacle, the SER spectra presented in this study were recorded on solid nanosilver plasmonic substrates developed in our laboratory, according to the procedure described in the materials section.

**Figure 1:**
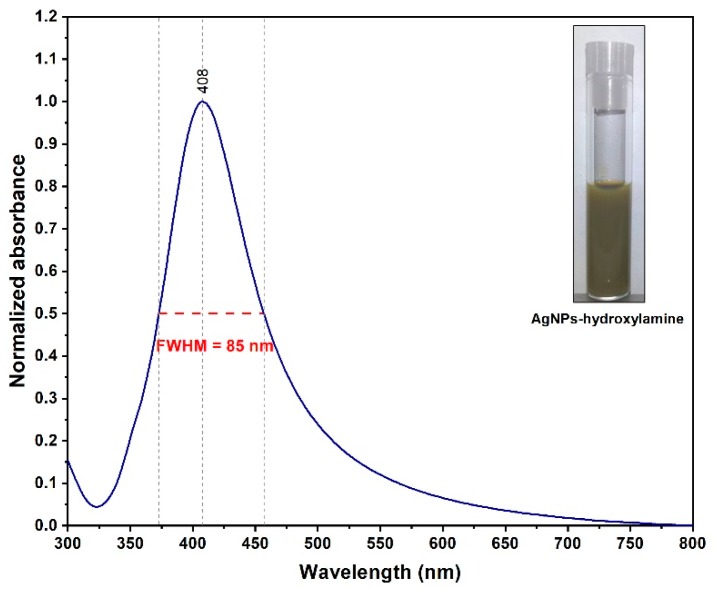
UV–VIS absorption spectrum of the filtered silver colloid. The inset presents an optical image of the concentrated colloidal solution. The full width at half maximum (FWHM) value is 85 nm.

The first step in the production of the solid plasmonic substrates was the synthesis of uniform spherical silver NPs. In order to remove all the secondary products resulted in the synthesis process and to improve NPs dispersity, by the end of the synthesis process the colloidal solutions have been purified and concentrated 10 times by means of Tangential Flow Filtering (TFF) [35]. The UV–VIS absorption spectrum of the filtered colloids is presented in Figure 1. A very narrow absorption peak located at 408 nm, having a full width half maximum (FWHM) value of 85 nm, can be observed. This is the first indication of a high degree of NPs monodispersity.

**Figure 2:**
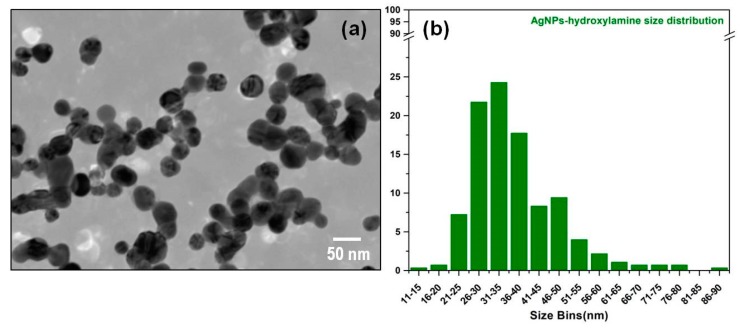
**Transmission Electron Microscopy** (TEM) image of the silver nanoparticles (**a**). Statistical analysis of nanoparticles diameters obtained from TEM images (**b**).

The TEM analysis presented in Figure 2 confirms the successful synthesis of spherical NPs. The statistical analysis of the acquired TEM images indicates a mean diameter of ~35 ± 5 nm, a value which is in good agreement with the UV–VIS absorption spectrum presented in Figure 1.

**Figure 3:**
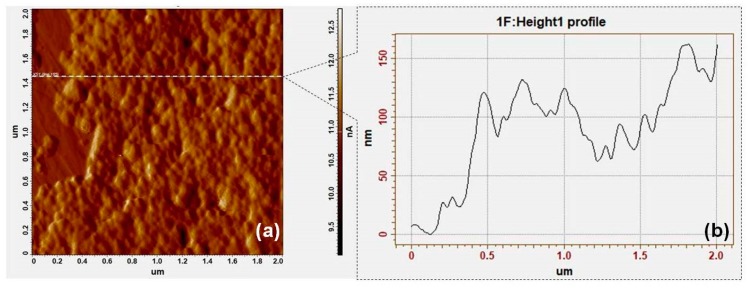
Topographic Atomic Force Microscopy (AFM) image of the solid plasmonic nanosilver substrate (**a**). Height profile of the substrate (**b**).

As it has been previously shown, SERS measurements performed on liquid saliva are dominated by the presence of large proteins [40,41]. In order to overcome these drawbacks, the SERS measurements presented in this study have been performed on saliva samples deposited on solid nanosilver substrates produced according to the procedure described in the Materials and Methods section. After their production, the substrate’s morphology was investigated by means of AFM measurements performed in semi-contact mode. A typical topographic image of the solid substrate is presented in Figure 3a. The solid substrate presents uniform areas created by a heat-induced self-assembling mechanism of purified AgNPs-hydroxylamine. The height of these areas is between ~70 nm and ~150 nm, as can be deduced from the height profile shown in Figure 3b. By considering the mean diameter of the NPs as 35 nm one can conclude that NPs organize in a 3D manner on top of the CaF_2_ glass slides. The presence of this type of large scale nanostructured plasmonic areas make them ideal substrates for SERS measurements on saliva, capable of generating reproducible and information-rich SERS signals.

**Figure 4:**
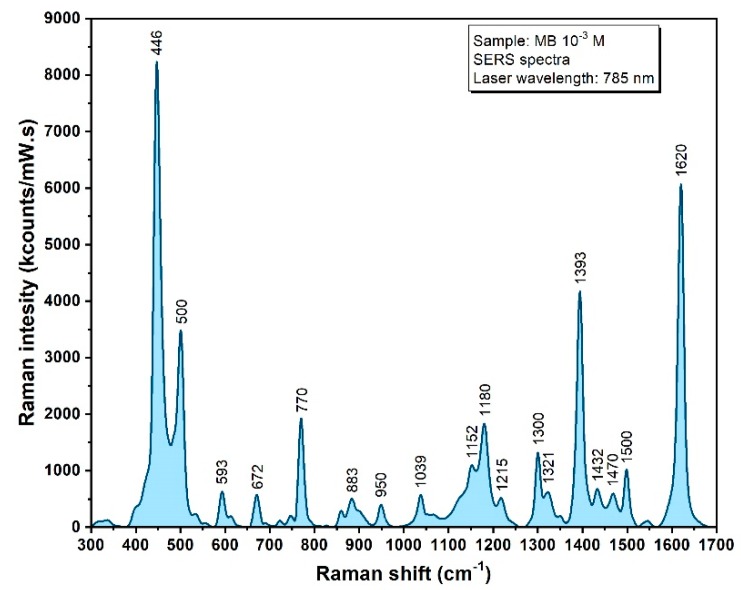
SERS spectrum of a dried solution of Methylene Blue (MB, 1 mM) recorded using a 785 nm excitation laser.

The substrates ability to enhance the Raman signals has been verified on a test molecule (Methylene Blue—MB), by employing a near infrared (NIR) excitation laser (λ = 785 nm) capable of reducing the fluorescence background. The mean spectrum of 25 signal acquisitions from different areas of the substrate is presented in Figure 4. The vast majority of MB specific vibrational bands are present, while being fully reproducible on whole scanned surfaces with very small variations in intensity [18,42]. For a proper quantitative assignment of these intensities, they are represented in kcounts/(mW·s) units.

### 3.2. Raman Spectrum of Saliva

The analysis of saliva at the molecular level can provide important information related to the pathological mechanisms of various organs within the body [41]. It has been reported that Raman spectroscopy of saliva can be used for narcotic usage detection, for cancer diagnostics, and in forensic medicine [40,43,44,45]. Saliva is a very complex solution that has a composition dependent on different factors such as lifestyle or circadian rhythm [46]. Whole saliva contains water as a dominant component (>90%), but organic and inorganic molecules with important biological roles are also present in different amounts. The organic components are mainly represented by proteins, polypeptides, immunoglobulins, lipids, vitamins, hormones, and organic acids. Among the inorganic components present in saliva, thiocyanate (SCN-) is of tremendous importance, taking part in the host defense mechanisms and acting as an antioxidant agent. SCN- participates as a substrate in the lactoperoxidase-driven catalytic reduction of hydrogen peroxide forming hypothiocyanous acid (HOSCN). HOSCN is a strong antimicrobial agent that removes or inhibits pathogens, but is tolerated by the host tissue [24]. Compared to other biofluids, saliva has higher concentrations of SCN- (0.5–3 mM) because of its necessity for antimicrobial protection [47]. The high concentration of SCN- in the oral cavity underscores its importance. Since SCN- can be utilized by peroxidases to generate HOSCN, it was hypothesized that elevated SCN- will predispose individuals to inflammation and disease [48,49]. Until now, the determination of physiological levels of salivary SCN- have been used to distinguish smokers from nonsmokers [49]. In order to evaluate its capacity to act as a possible biomarker for IR irradiation evaluation, we have performed a combined Raman/SERS analysis on salivary samples collected from ten children (before and after IR irradiation).

**Figure 5:**
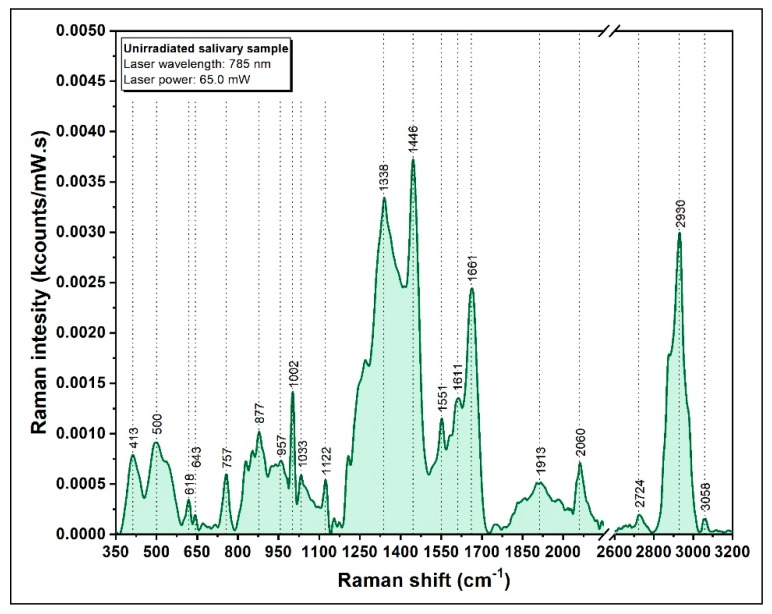
A typical Raman spectrum of dried saliva, recorded using a 785 nm laser.

In Figure 5, a typical Raman spectrum of dried saliva, acquired using an excitation wavelength of 785 nm, is presented. As expected, the spectrum is dominated by the organic biomolecules present in saliva.

The vibrational bands located at 1446 and 1661 cm^−1^ can be associated with the presence of lysozymes with the observation that the 1661 cm^−1^ peak is also characteristic for protein specific Amide I group [50,51]. The strong Raman peak around 1002 cm^−1^ can be attributed to phenylalanine contained in amylase, lipase, or other proteins [52,53]. The presence of the 1450 cm^−1^ peak has been detected in different proteins (e.g., the glycoproteins forming the mucus), but also in collagen and lipids [54,55,56]. The peaks in the low wavenumber range of 350–550 cm^−1^ could be attributed to the presence of saccharides such as mucopolysaccharides of the mucus [57,58]. The peak at 2060 cm^−1^ is specific for saliva and according to Farquharson et al. it can be attributed to thiocyanate [45]. One of the major advantages of this vibrational peak is the fact that it does not overlap with any other biomolecular vibrations being very specific for SCN-. As a direct consequence of its biological role and ease of detection in Raman/SER spectroscopy, this peak will be used for evaluating the biological effects of IR on children.

### 3.3. SERS Spectrum of Saliva

In order to reduce the influence that proteins and large biomolecules have on vibrational spectra and to enhance the chances for specific biomarker detection in saliva, SERS measurements have been performed on dried salivary samples collected from the children included in this study, before and after IR irradiation, by using the solid plasmonic substrates described above. 

#### 3.3.1. SERS Spectrum of Healthy Donors

**Figure 6:**
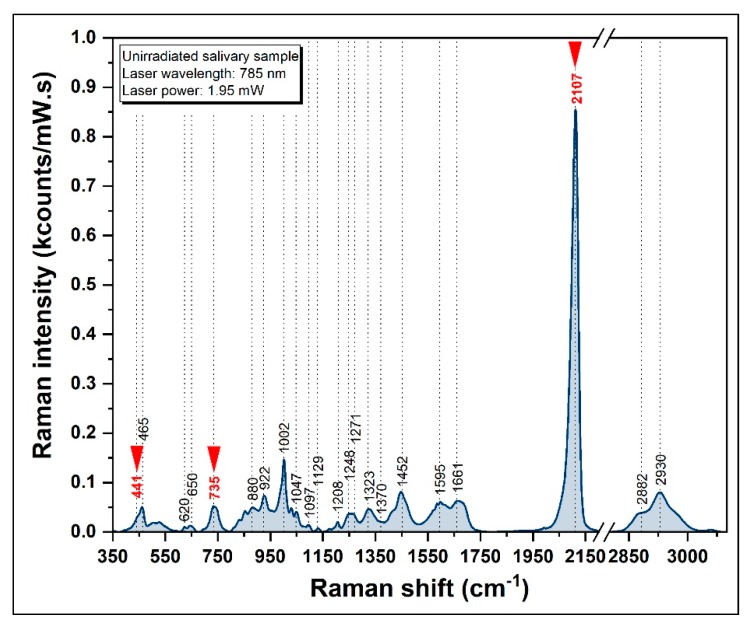
A typical SER spectrum of saliva (λ = 785 nm). The red arrows indicate the thiocyanate specific vibrational bands.

A typical salivary SER spectrum of saliva, acquired using a 785 nm laser and a solid silver plasmonic substrate, is presented in Figure 6. The spectroscopic signature of saliva is consistent with other studies, confirming that the intensities of the bands found in saliva are proportional to their concentration [40,59].

The spectrum is completely different with respect to the previously recorded Raman spectrum obtained on the same saliva sample by being dominated by several vibrational bands. Among these, the 2107 cm^−1^ peak (specific for SCN-) is by far the most intense one. The other two peaks of SCN- are present in the spectrum (441 and 735 cm^−1^). It has been shown that these vibrational modes correspond to bending and stretching vibrations of SCN- [60,61,62]. The SERS spectra of pure thiocyanate at different physiological concentrations ranging between 0.1 mM and 3 mM, recorded on the same substrate and using identical experimental conditions are presented in Figure S1. In the case of saliva, the 2107 cm^−1^ peak is almost ten times more intense than any other peak present in the SER spectrum. As a direct consequence of this fact, very recently the use of SCN- as a biomarker for the detection and identification of liquid and dried saliva has been reported [63]. The presence of other components, such as proteins and amino acids, could also be detected in the SER spectrum presented in Figure 6. Although their intensities are greater than the Raman spectrum, the most remarkable increase is observed in the case of the vibrational bands assigned to SCN-. This is probably due to a higher affinity of SCN- molecules for metallic surfaces and to the substrates capacity to enhance the Raman signal of this very small biomolecule. A tentative assignment of the vibrational bands shown in Figure 6 is provided in Table 1.

#### 3.3.2. SERS Spectrum of Irradiated Salivary Samples

**Figure 7:**
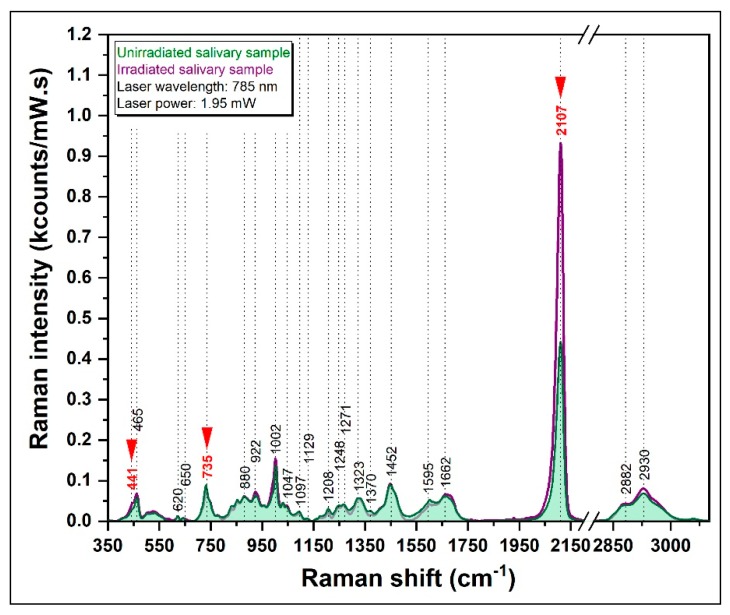
Superposition of the SER spectra recorded before (green spectra) and after irradiation (purple spectra) for one of the patients involved in this study. The spectra have been recorded using an excitation wavelength of 785 nm.

The Raman vibrations in the 350–3000 cm^−1^ spectral region were also analyzed for irradiated salivary samples. The results were superposed with those recorded before irradiation and are presented in Figure 7, in the case of the same sample. The results have been confirmed in the case of all other nine samples. The intensity variations of the 2107 cm^−1^ vibrational peak before/after irradiation for all the 10 samples included in this study are presented in Table S2.

As can be seen in the figure, the spectrum of saliva differs before and after irradiation, but not in a uniform way. The only peak that showed a greater intensity in all the samples of the irradiated group is 2107 cm^−1^, which can be assigned to the -C≡N stretching of thiocyanate, most probably due to an increase of SCN- concentration in saliva induced by irradiation. It should be mentioned that in the case of all saliva samples the intensity of the 2107 cm^−1^ vibrational peak increased. However, this increase was not uniform. 

The SCN- half-life in saliva is 10–14 days [63]. In this study, we have shown that a sudden increase of its salivary concentration (observed in a very short period of time with respect to the half-life) as a consequence of irradiation can be experimentally proven by means of SERS. This represents strong experimental evidence that SCN- can be successfully used as a low-dose IR biomarker, but further quantitative analysis is still needed.

## 4. Conclusions

SCN’s antioxidative capacity to scavenge oxidizing agents has never been directly proven experimentally until now. In this study we showed by means of SERS that an immediate increase of vibrational bands assigned to SCN- can be detected as a result of IR interaction with the oral cavity. This is a direct proof of the change of salivary SCN- concentration, probably as a result of the activation of salivary antioxidant system. Even if this change is not uniform for all samples, it is notable that this increase has been observed in the case of all investigated saliva samples presented in this study, after a very short period of time following the irradiation process (less than 30 min). These results could not be evidenced by standard Raman, but have been proven only by means of SERS measurements performed on a nanosilver solid plasmonic substrate developed in our laboratory, probably due to the high affinity of SCN- molecule for the silver surface. This work experimentally proves the major role played by SCN- in the defense mechanism and could consecrate its use as a valuable tool for pediatric radiation dose optimization. Nevertheless, these preliminary data suggest its possible use in other scientific domains of paramount interest, such as radiation and cancer research.

## Figures and Tables

**Table 1:** Assignment of the vibrational peaks recorded in the SERS spectra of human saliva.

Raman Band (cm^−1^)	Vibrational Mode	Assignment
441	S−C≡N bending vibration	Thiocyanate
620	C-C twisting mode	Phenylalanine
650	C-C twisting mode	Phenylalanine
735	C−S stretching vibration	Thiocyanate
880	v (C-C)	Hydroxyproline
1002	ν_S_ (C-C)	Phenylalanine
1047	ν (C-O), ν (C-N)	Proteins
1208	ν (C-C6H5)	Tryptophan
1452	δ (C-H)	Collagen, lipids
1661	ν (C=C)	Amide I
2107	−C≡N stretching vibration	Thiocyanate

## Supplementary Materials

The following are available online at https://www.mdpi.com/2075-4418/9/3/101/s1.

## References

[1] Pernot E., Hall J., Baatout S., Benotmane M.A., Blanchardon E., Bouffler S., El Saghire H., Gomolka M., Guertler A., Harms-Ringdahl M. (2012). Ionizing radiation biomarkers for potential use in epidemiological studies. Mutat. Res..

[2] Jennings F.L. (1966). Mammalian Radiation Lethality: A Disturbance in Cellular Kinetics. Jama J. Am. Med. Assoc..

[3] Eric J., Hall A.J.G. (2000). Radiobiology for the Radiologist.

[4] Fazel R., Krumholz H.M., Wang Y., Ross J.S., Chen J., Ting H.H., Shah N.D., Nasir K., Einstein A.J., Nallamothu B.K. (2009). Exposure to Low-Dose Ionizing Radiation from Medical Imaging Procedures. N. Engl. J. Med..

[5] Oenning A.C., Jacobs R., Pauwels R., Stratis A., Hedesiu M., Salmon B., DIMITRA Research Group (2018). Cone-beam CT in paediatric dentistry: DIMITRA project position statement. Pediatr. Radiol..

[6] Kapila S.D., Nervina J.M. (2015). CBCT in orthodontics: Assessment of treatment outcomes and indications for its use. Dentomaxillofacial Radiol..

[7] Hujoel P., Hollender L., Bollen A.-M., Young J.D., McGee M., Grosso A. (2008). Head-and-neck organ doses from an episode of orthodontic care. Am. J. Orthod. Dentofac. Orthop..

[8] Kreuzer M., Auvinen A., Cardis E., Durante M., Harms-Ringdahl M., Jourdain J.R., Madas B.G., Ottolenghi A., Pazzaglia S., Prise K.M. (2018). Multidisciplinary European Low Dose Initiative (MELODI): Strategic research agenda for low dose radiation risk research. Radiat. Environ. Biophys..

[9] Pernot E., Cardis E., Badie C. (2014). Usefulness of Saliva Samples for Biomarker Studies in Radiation Research. Cancer Epidemiol. Biomark. Prev..

[10] Liu J., Duan Y. (2012). Saliva: A potential media for disease diagnostics and monitoring. Oral Oncol..

[11] Theodorakou C., Walker A., Horner K., Pauwels R., Bogaerts R., Jacobs Dds R., SEDENTEXCT Project Consortium (2012). Estimation of paediatric organ and effective doses from dental cone beam CT using anthropomorphic phantoms. Br. J. Radiol..

[12] Bonne N.J., Wong D.T. (2012). Salivary biomarker development using genomic, proteomic and metabolomic approaches. Genome Med..

[13] Vandenabeele P. (2013). Practical Raman Spectroscopy: An Introduction.

[14] Grasselli J. (1981). Chemical Applications of Raman Spectroscopy.

[15] Simon I., Hedesiu M., Virag P., Salmon B., Tarmure V., Baciut M., Bran S., Jacobs R., Falamas A. (2019). Raman Micro-Spectroscopy of Dental Pulp Stem Cells: An Approach to Monitor the Effects of Cone Beam Computed Tomography Low-Dose Ionizing Radiation. Anal. Lett..

[16] Bonifacio A., Cervo S., Sergo V. (2015). Label-free surface-enhanced Raman spectroscopy of biofluids: Fundamental aspects and diagnostic applications. Anal. Bioanal. Chem..

[17] Schlücker S. (2011). Surface Enhanced Raman Spectroscopy: Analytical, Biophysical and Life Science Applications.

[18] Leopold N., Bernhard L. (2003). A New Method for Fast Preparation of Highly Surface-Enhanced Raman Scattering (SERS) Active Silver Colloids at Room Temperature by Reduction of Silver Nitrate with Hydroxylamine Hydrochloride. J. Phys. Chem. B.

[19] Connolly J.M., Davies K., Kazakeviciute A., Wheatley A.M., Dockery P., Keogh I., Olivo M. (2016). Non-invasive and label-free detection of oral squamous cell carcinoma using saliva surface-enhanced Raman spectroscopy and multivariate analysis. Nanomed. Nanotechnol. Biol. Med..

[20] Feng S., Huang S., Lin D., Chen G., Xu Y., Li Y., Huang Z., Pan J., Chen R., Zeng H. (2015). Surface-enhanced Raman spectroscopy of saliva proteins for the noninvasive differentiation of benign and malignant breast tumors. Int. J. Nanomed..

[21] Qiu S., Xu Y., Huang L., Zheng W., Huang C., Huang S., Lin J., Lin D., Feng S., Chen R. (2016). Non-invasive detection of nasopharyngeal carcinoma using saliva surface-enhanced Raman spectroscopy. Oncol. Lett..

[22] Li X., Yang T., Lin J. (2012). Spectral analysis of human saliva for detection of lung cancer using surface-enhanced Raman spectroscopy. J. Biomed. Opt..

[23] Stefancu A., Badarinza M., Moisoiu V., Iancu S.D., Serban O., Leopold N., Fodor D. (2019). SERS-based liquid biopsy of saliva and serum from patients with Sjögren’s syndrome. Anal. Bioanal. Chem..

[24] Chandler J.D., Day B.J. (2012). THIOCYANATE: A potentially useful therapeutic agent with host defense and antioxidant properties. Biochem. Pharm..

[25] Olea F., Parras P. (1992). Determination of Serum Levels of Dietary Thiocyanate. J. Anal. Toxicol..

[26] Hassan S.S.M., Hamza M.S.A., Kelany A.E. (2007). A novel spectrophotometric method for batch and flow injection determination of cyanide in electroplating wastewater. Talanta.

[27] Michigami Y., Fujii K., Ueda K., Yamamoto Y. (1992). Determination of thiocyanate in human saliva and urine by ion chromatography. Analyst.

[28] Blount B.C., Özpinar A., Alwis K.U., Caudill S.P., Gillespie J.R. (2008). Perchlorate, Nitrate, Thiocyanate, and Iodide Levels in Chicken Feed, Water, and Eggs from Three Farms. J. Agric. Food Chem..

[29] Yang L., Yu H., Wang Y. (2010). Rapid and Simultaneous Determination of Tetrafluoroborate, Thiocyanate and Hexafluorophosphate by High-Performance Liquid Chromatography Using a Monolithic Column and Direct Conductivity Detection. Anal. Sci..

[30] Hou T., Liu Y., Xu L., Wu Y., Ying Y., Wen Y., Guo X., Yang H. (2017). Au dotted magnetic graphene sheets for sensitive detection of thiocyanate. Sens. Actuators B Chem..

[31] Tsuge K., Kataoka M., Seto Y. (2000). Cyanide and Thiocyanate Levels in Blood and Saliva of Healthy Adult Volunteers. J. Health Sci..

[32] Aggarwal A., Keluskar V., Goyal R., Dahiya P. (2013). Salivary thiocyanate: A biochemical indicator of cigarette smoking in adolescents. Oral Health Prev. Dent..

[33] Marcu M., Hedesiu M., Salmon B., Pauwels R., Stratis A., Oenning A.C.C., Cohen M.E., Jacobs R., Baciut M., Roman R. (2018). Estimation of the radiation dose for pediatric CBCT indications: A prospective study on ProMax3D. Int. J. Paediatr. Dent..

[34] Munro C.L., Grap M.J., Jablonski R., Boyle A. (2006). Oral health measurement in nursing research: State of the science. Biol. Res. Nurs..

[35] Dorney K.M., Baker J.D., Edwards M.L., Kanel S.R., O’Malley M., Pavel Sizemore I.E. (2014). Tangential Flow Filtration of Colloidal Silver Nanoparticles: A “Green” Laboratory Experiment for Chemistry and Engineering Students. J. Chem. Educ..

[36] Thanu D.P.R., Srinadhu E.S., Zhao M., Dole N.V., Keswani M. (2019). Fundamentals and Applications of Plasma Cleaning. Developments in Surface Contamination and Cleaning: Applications of Cleaning Techniques.

[37] Bonifacio A., Dalla Marta S., Spizzo R., Cervo S., Steffan A., Colombatti A., Sergo V. (2014). Surface-enhanced Raman spectroscopy of blood plasma and serum using Ag and Au nanoparticles: A systematic study. Anal. Bioanal. Chem..

[38] Gebauer J.S., Malissek M., Simon S., Knauer S.K., Maskos M., Stauber R.H., Peukert W., Treuel L. (2012). Impact of the Nanoparticle–Protein Corona on Colloidal Stability and Protein Structure. Langmuir.

[39] Walkey C.D., Chan W.C.W. (2012). Understanding and controlling the interaction of nanomaterials with proteins in a physiological environment. Chem. Soc. Rev..

[40] Virkler K., Lednev I.K. (2010). Forensic body fluid identification: The Raman spectroscopic signature of saliva. Analyst.

[41] Gonchukov S., Sukhinina A., Bakhmutov D., Minaeva S. (2012). Raman spectroscopy of saliva as a perspective method for periodontitis diagnostics. Laser Phys. Lett..

[42] Xiao G.-N., Man S.-Q. (2007). Surface-enhanced Raman scattering of methylene blue adsorbed on cap-shaped silver nanoparticles. Chem. Phys. Lett..

[43] Anyu C., Lin H., Jinghua L., ZiJian C., Yi J., Dian Q., Xun G., Chunwei L., Wen H., Hong W. (2009). Detecting Narcotic Usage Using Surface-Enhanced Raman Spectroscopy on Saliva Samples. Proceedings of the World Congress on Medical Physics and Biomedical Engineering.

[44] Kah J.C.Y., Kho K.W., Lee C.G.L., James C., Sheppard R., Shen Z.X., Soo K.C., Olivo M.C. (2007). Early diagnosis of oral cancer based on the surface plasmon resonance of gold nanoparticles. Int. J. Nanomed..

[45] Farquharson S., Shende C., Inscore F.E., Maksymiuk P., Gift A. (2005). Analysis of 5-fluorouracil in saliva using surface-enhanced Raman spectroscopy. J. Raman Spectrosc..

[46] de Almeida P.D.V., Grégio A.M.T., Machado M.A.N., de Lima A.A.S., Azevedo L.R. (2008). Saliva composition and functions: A comprehensive review. J. Contemp. Dent. Pract..

[47] Ashby M.T. (2008). Inorganic Chemistry of Defensive Peroxidases in the Human Oral Cavity. J. Dent. Res..

[48] Wang Z., Nicholls S.J., Rodriguez E.R., Kummu O., Hörkkö S., Barnard J., Reynolds W.F., Topol E.J., DiDonato J.A., Hazen S.L. (2007). Protein carbamylation links inflammation, smoking, uremia and atherogenesis. Nat. Med..

[49] Morgan P.E., Pattison D.I., Talib J., Summers F.A., Harmer J.A., Celermajer D.S., Hawkins C.L., Davies M.J. (2011). High plasma thiocyanate levels in smokers are a key determinant of thiol oxidation induced by myeloperoxidase. Free Radic. Biol. Med..

[50] Chen M.C., Lord R.C., Mendelsohn R. (1974). Laser-excited Raman spectroscopy of biomolecules. V. Conformational changes associated with the chemical denaturation of lysozyme. J. Am. Chem. Soc..

[51] Remmele R.L., McMillan P., Bieber A. (1990). Raman spectroscopic studies of hen egg-white lysozyme at high temperatures and pressures. J. Protein Chem..

[52] Stein E.A., Junge J.M., Fisher E.H. (1960). The amino acid composition of alpha-amylase from Aspergillus oryzae. J. Biol. Chem..

[53] Bianchetta J.D., Bidaud J., Guidoni A.A., Bonicel J.J., Rovery M. (1979). Porcine pancreatic lipase. Sequence of the first 234 amino acids of the peptide chain. Eur. J. Biochem..

[54] Vanni S. (2003). Raman Chemical Imaging Provides Rapid, Non-Invasive and Reagentless Biothreat Detection.

[55] Kalasinsky K.S., Hadfield T., Shea A.A., Kalasinsky V.F., Nelson M.P., Neiss J., Drauch A.J., Vanni G.S., Treado P.J. (2007). Raman Chemical Imaging Spectroscopy Reagentless Detection and Identification of Pathogens: Signature Development and Evaluation. Anal. Chem..

[56] Stiufiuc R., Iacovita C., Stiufiuc G., Florea A., Achim M., Lucaciu C.M. (2015). A new class of pegylated plasmonic liposomes: Synthesis and characterization. J. Colloid Interface Sci..

[57] De Gelder J., De Gussem K., Vandenabeele P., Moens L. (2007). Reference database of Raman spectra of biological molecules. J. Raman Spectrosc..

[58] Barrett T.W. (1981). Laser Raman spectra of mono-, oligo- and polysaccharides in solution. Spectrochim. Acta Part. Mol. Spectrosc..

[59] Khaustova S., Shkurnikov M., Tonevitsky E., Artyushenko V., Tonevitsky A. (2010). Noninvasive biochemical monitoring of physiological stress by Fourier transform infrared saliva spectroscopy. Analyst.

[60] Bron M., Holze R. (1999). The adsorption of thiocyanate ions at gold electrodes from an alkaline electrolyte solution: A combined in situ infrared and raman spectroscopic study. Electrochimica Acta.

[61] Wu L., Wang Z., Zong S., Cui Y. (2014). Rapid and reproducible analysis of thiocyanate in real human serum and saliva using a droplet SERS-microfluidic chip. Biosens. Bioelectron..

[62] Yang Q., Liang F., Wang D., Ma P., Gao D., Han J., Li Y., Yu A., Song D., Wang X. (2014). Simultaneous determination of thiocyanate ion and melamine in milk and milk powder using surface-enhanced Raman spectroscopy. Anal. Methods.

[63] Wong M. (2017). Surface-Enhanced Raman Spectroscopy for Forensic Analysis of Human Saliva. https://open.bu.edu/handle/2144/23997.

