# Supplementary material for: The Effects of Low-Dose Irradiation on Human Saliva: A Surface-Enhanced Raman Spectroscopy Study
Source: Diagnostics (Basel). 2019 Aug 22;9(3):101. doi: 10.3390/diagnostics9030101 (PMC6787699; doi:10.3390/diagnostics9030101)
Supplement: Supplementary file 1 [file diagnostics-09-00101-s001.pdf]

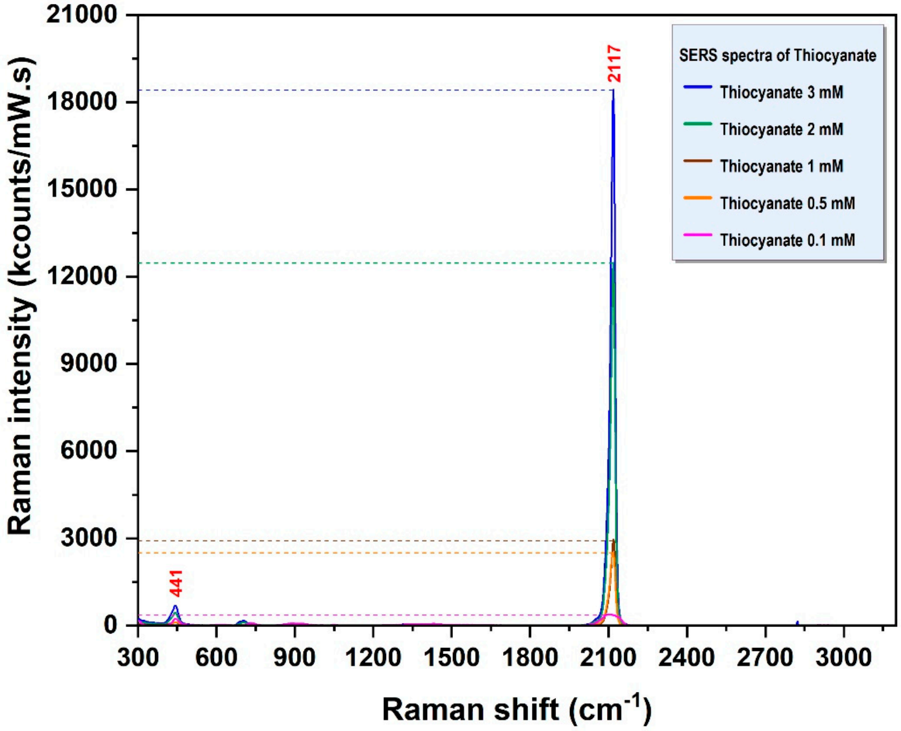

Figure S1: SER spectra of pure thiocyanate solutions of different physiological concentrations ranging between 0.1–3 mM. The spectra have been recorded using the here proposed solid silver plasmonic substrates and an excitation wavelength of 785 nm.

Table S1: The variables of the patients irradiated with a large field of view CBCT and the duration of CBCT exposure. The effective irradiation dose has been calculated using a Monte Carlo simulation method.

Probe	Age	Sex	kV	mA x seconds	Effective dose (μSv)
**1**	13	M	83	4 x 13.6	174.3
**2**	12	F	83	4 x 13.559	217.9
**3**	17	F	85	5.6 x 13.572	256.1
**4**	13	F	83	4 x 13.619	174.3
**5**	16	F	83	4 x 13.648	174.3
**6**	15	M	83	4 x 13.428	174
**7**	13	F	83	4 x 13.690	174.4

**8**	15	F	83	4 x 13.712	175.1
**9**	14	F	83	4 x 13.721	175.2
**10**	13	M	83	4 x 13.602	174.2

Table S2: The effective dose of irradiation and the intensities variations of the 2107 cm^{-1} vibrational peak before/after irradiation for all the 10 samples introduced in this study.

**Probe**	**Effective dose (μSv)**	**Intensity of 2107 cm^{-1} peak (kcts mW^{-1} s^{-1}) before/after**
**1**	174.3	0.320/0.695
**2**	217.9	0.442/0.933
**3**	256.1	0.650/0.698
**4**	174.3	0.559/0.681
**5**	174.3	0.558/0.599
**6**	174	0.421/0.756
**7**	174.4	0.390/0.681
**8**	175.1	0.321/0.625
**9**	175.2	0.401/0.728
**10**	174.2	0.511/0.634
